# Implementation of a Community Transport Strategy to Reduce Delays in Seeking Obstetric Care in Rural Mozambique

**DOI:** 10.9745/GHSP-D-20-00511

**Published:** 2021-03-15

**Authors:** Felizarda Amosse, Helena Boene, Mai-Lei Woo Kinshella, Sharla Drebit, Sumedha Sharma, Prestige Tatenda Makanga, Anifa Valá, Laura A. Magee, Peter von Dadelszen, Marianne Vidler, Esperança Sevene, Khátia Munguambe, the Community Level Interventions for Pre-eclampsia (CLIP) Working Group

**Affiliations:** a Centro de Investigação em Saúde da Manhiça, Maputo, Mozambique.; b Department of Obstetrics and Gynaecology, University of British Columbia, Vancouver, Canada.; c Place Alert Labs, Surveying, and Geomatics Department, Midlands State University, Gweru, Zimbabwe.; d School of Life Course Sciences, King's Collage London, Strand, London, UK.; e Faculdade de Medicina, Universidade Eduardo Mondlane, Maputo, Mozambique.

## Abstract

**Introduction:**

Delays due to long distances to health facilities, poor road infrastructure, and lack of affordable transport options contribute to the burden of maternal deaths in Mozambique. This study aimed to assess the implementation and uptake of an innovative community-based transport program to improve access to emergency obstetric care in southern Mozambique.

**Methods:**

From April 2016 to February 2017, a community transport strategy was implemented as part of the Community Level Interventions for Pre-eclampsia Trial. The study aimed to reduce maternal and perinatal mortality and morbidity by 20% in intervention clusters in Maputo and Gaza Provinces, Mozambique, by involving community health workers in the identification and referral of pregnant and puerperal women at risk. Based on a community-based participatory needs assessment, the transport program was implemented with the trial. Demographics, conditions requiring transportation, means of transport used, route, and outcomes were collected during implementation. Data were entered into a REDCap database.

**Results:**

Fifty-seven neighborhoods contributed to the needs assessment; of those, 13 (23%) implemented the transport program. Neighborhoods were selected based on their expression of interest and ability to contribute financially to the program (US$0.33 per family per month). In each selected neighborhood, a community management committee was created, training in small-scale financial management was conducted, and monitoring tools were provided. Twenty people from 9 neighborhoods benefited from the transport program, 70% were pregnant and postpartum women.

**Conclusion:**

These results demonstrate that it was feasible to implement a community-based transport program with no external input of vehicles, fuel, personnel, and maintenance. However, high cost and a lack of acceptable transport options in some communities continue to impede access to obstetric health care services and the ability for timely follow-up. When strengthening capacities of community health workers to promptly assist and refer emergency cases, it is crucial to encourage local transport programs and transportation infrastructure among minimally resourced communities to support access and engagement with health systems.

## Abstract (precis)

Encouraging local transport programs and transport infrastructure in poorly-resourced communities can help improve community access and strengthen engagement with health systems. Mobilizing community resources and leadership to implement a community-based transport scheme in rural Mozambique to support referrals to health facilities can help improve maternal and child health outcomes.

## Teaser

**Key Findings:**

- It was feasible to implement a community-based transport program in rural areas with no external input of vehicles, fuel, personnel, or maintenance. However, lack of available transporters, barriers to fulfill requirements to opening and managing bank accounts, inequities in affordability of the scheme, and tensions regarding views on how to manage funds were implementation challenges identified that require further support.
- Positive impacts of program implementation included streamlining the process of identifying transport options and collaborators in the community, pre-negotiating travel prices to increase affordability, raising community attention to the role of transport in reducing delays in health care seeking, and raising awareness on the importance of early recognition of warning signs and prompt care seeking.

**Key Implications:**

- Our community-based transport program uniquely emphasized community ownership and leadership at all stages. Decisions were built by consensus within each community, which allowed for flexibility to local conditions and supported local skill and capacity building for financial management.
- Transport schemes cannot be implemented in isolation from the wider health system strengthening efforts. This scheme used existing referral pathways rather than creating entirely new processes to help sustainability.

Resumo em português no final do artigo.

## BACKGROUND

Annually, approximately 18 million women in Africa give birth at home without medical assistance. If complications arise, transport to a health facility is often unavailable or of poor quality.^1^ In Mozambique, the maternal mortality rate is 289 deaths per 100,000 live births in 2017^2^ and is associated with hemorrhage, hypertensive disorders, and sepsis.^3^ The 3 delays in seeking, accessing, and receiving appropriate and quality maternal health care contribute to many of these deaths.^1^^,^^4^ Maternal deaths in Mozambique are concentrated in rural communities where poverty rates are high. Rural communities have limited access to health services because of distance, lack of transport, and poor roads, contributing to delays for pregnant and postpartum women accessing care.^5^ These factors contribute to delays for pregnant and postpartum women accessing care, which is particularly dangerous in emergencies where every delay increases the risk of stillbirth, neonatal, or maternal death. A study from Maputo and Gaza Provinces in Southern Mozambique found that owning a private vehicle was rare, and rural women walked on average half an hour—up to 2 hours for the most isolated communities—to reach the nearest main road where public transport could be found.^6^ Increased isolation was significantly associated with higher rates of adverse maternal and perinatal outcomes.^6^

Rural communities have limited access to health services because of distance, lack of transport, and poor roads, contributing to delays for pregnant and postpartum women accessing care.

Community health worker (CHW) programs support delivering maternal and child health care in resource-limited settings and provide a valuable link between communities and facility-based health services.^7^ The 2018 World Health Organization (WHO) guidelines on health policy and system support to optimize CHW programs highlighted the need to better embed CHWs into health systems^8^ and for CHWs to provide timely and appropriate referral from the community to the health facility.^9^ As such, access to emergency transport for pregnant and postpartum women is essential in preventing maternal deaths.^10^ A study in Ghana emphasized concerns about unreliable transport to seek health care, especially in emergencies, and highlighted the need for policies to solve rural transport problems to improve maternal health.^11^ With increasing attention to the mobilization of local resources to improve access to services, a community-based action research study in Haiti suggested that in addition to the 3 delays, there was a fourth delay related to mobilization of human and financial resources, such as community transport programs, to reduce maternal deaths.^12^ In a systematic review of studies conducted in Bangladesh, Ghana, India, Malawi, Nepal, Nigeria, Sierra Leone, and Tanzania, community loans for emergency transport increased the utilization of health facilities for delivery and emergency obstetric care.^10^

Given that delays reaching health facilities in case of emergency care in low- and middle-income countries can be reduced through the implementation of transport programs,^13^ there is a need to research questions on whether and how best these programs can operate under real-life situations taking into account the existing community health and primary health care (PHC) programs and respective referral systems. Such programs can include direct provision of transport for pregnant women in need of emergency obstetric care in an appropriate health care facility.^14^ This study aimed to describe the implementation process of a community transport program to reduce delays in accessing emergency obstetric care in southern Mozambique.

## METHODS

The community transport program was embedded within the larger Community-Level Interventions for Pre-eclampsia (CLIP) in Mozambique Trial (National Clinical Trials #01911494), which aimed to reduce maternal and perinatal mortality and morbidity by strengthening CHWs' capacity to identify high-risk pregnancies and refer them to the health facility when needed.^14^ The CHWs in the CLIP Trial, known as agentes polivalentes elementares in Mozambique, belonged to the existing CHW program and received extra training as part of the trial. In turn, the trial was aligned with the existing referral system between the community and health facilities, whereby CHWs transfer patients that require higher levels of assistance by using referral slips that record the date, person's name, age, residence, referral facility, reported signs and symptoms, and first aid or care provided, and CHW name.

Formative research preceding the CLIP Trial, which used an ethnographic approach to investigate the problem of access to care, revealed that the lack of transport was a major contributor to poor referrals and hence low access to health care.^5^ In the same study, accounts from women of reproductive age, pregnant women, household decision makers, and health care providers in these communities revealed that even where transport was available in the form of minibuses or agricultural tractors, it was restricted to main roads or prohibitively expensive to arrange privately on a case-by-case basis for most women.^5^ Consequently, many residents had to walk long distances to access transport—a challenge during obstetric emergencies—or simply did not travel to the health facilities. Although ambulances were available to support referrals between facilities, there was no formal structured system providing transport from the community to health centers, mainly because of the physical distance between the communities and the health facilities. The transport program was designed to help facilitate the timely referral of pregnant women with obstetric emergencies to the nearest primary health facilities.

The transport program was designed to help facilitate the timely referral of pregnant women with obstetric emergencies to the nearest primary health facility.

### Study Area

**FIGURE 1:**
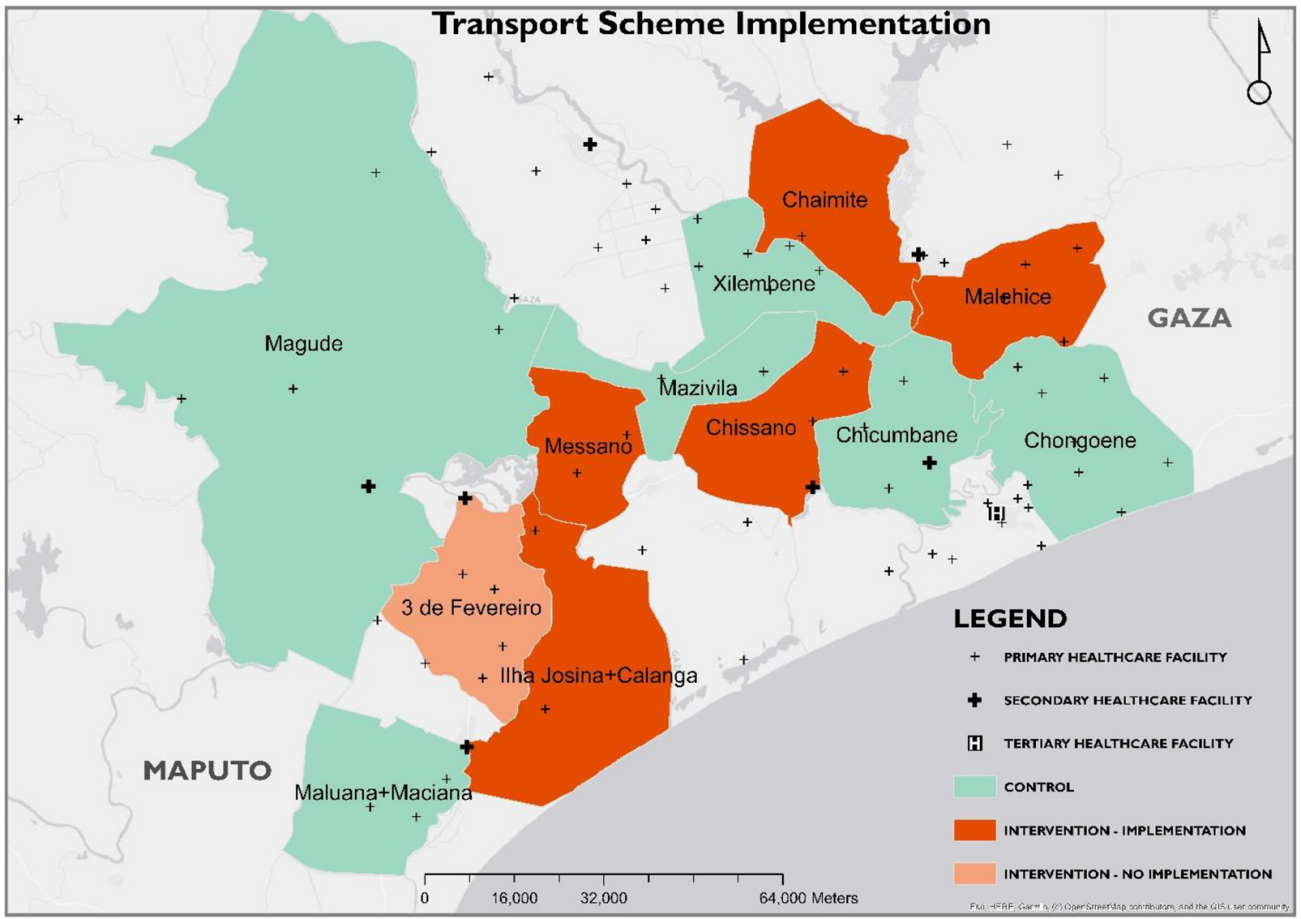
Map Showing Study Areas Where Community Transport Program to Improve Access to Emergency Obstetric Case Was Implemented, Southern Mozambique

The study area included Malehice, Chaimite, Chissano, Messano, Três de Fevereiro, Ilha Josina, and Calanga administrative posts from Maputo and Gaza Provinces in southern Mozambique (Figure 1), which were also part of the CLIP Trial. The study area is largely rural with agriculture, fishing, cattle breeding, and informal trade as the main resident income sources. During the rainy season from approximately November to March, some areas are severely affected by road blocks due to floods, particularly Ilha Josina and Calanga.^15^

### Program Context and Procedures

A community-based participatory needs assessment was conducted to inform the community transport plan and to support the creation of a community fund to cover transport costs. While community financing programs to support transport for emergency referral have been used in other areas in sub-Saharan Africa,^16^ this concept was novel to this region. There were existing microfinance programs for non-health purposes in the communities, so members were familiar with the practice.

As part of the rapport-building stage, contacts were first made with the community chief in each administrative post to obtain permission for the proposed activities. Subsequently, neighborhood chiefs (known as secretários dos bairros) were contacted to support scheduling of the activities with community members. The first activity consisted of 3 rounds of meetings: initial meetings to assess needs and raise awareness about the community transport program, mobilization meetings with interested communities to prepare for implementation, and follow-up meetings with communities who implemented the program. Community meetings, as part of community engagement activities for the CLIP Trial, included pregnant women, women of reproductive age, partners and husbands, mothers and mothers-in-law, and the community in general and were conducted at the círculos (the usual community gathering location). These meetings included discussions regarding launching the program; encouraging community contributions to the fund; sharing the list of transporters and management committee members; and presenting updates on uses, finances, and savings within the transport program. These community meetings occurred throughout the project and most meetings were conducted in Changana, the predominant local language. During the phase-out stage of the CLIP trial, a final round of community meetings was held to reflect upon the program achievements, and involved CHWs, selected community members, PHC facility staff, owners of the transport program vehicles and community leaders.

### Data Collection

Assessment of the transport program implementation utilized a mixed-methods approach. Both quantitative and qualitative data were collected using structured forms (referred to as logs). Quantitative data included demographic characteristics of meeting attendees and information associated with the management of funds and qualitative data included information on all medical complaints that required transport, transport methods used, transport users' and stakeholders' testimonials. Meeting details, including location, number of participants, and their backgrounds and messages discussed were also captured, including facilitator reflections and community feedback.

Data collection was conducted by a team comprising a social scientist, a community liaison officer, 3 mobilizers, and 4 health activists employed by the CLIP Trial, separate from CHWs in the neighborhoods. All data collectors were fluent in both Portuguese and Changana.

### Data Management and Analysis

All data were sent to the Manhiça Health Research Center for data entry to a REDCap database (Nashville, TN, USA).^17^ Before data entry, all logs were checked for quality by study team members who conducted data collection. Missing data, outliers, and discrepancies were queried to maximize data integrity. Data analyses were performed using RStudio software version 3.4.1 (RStudio Inc, Boston, MA, USA) to generate frequency distributions of categorical variables. The analysis separated general community meetings and meetings where transport issues were discussed, the latter of which is the focus of this article. Demographic characteristics of the participants and the study variables of interest are presented using descriptive statistics. Qualitative data was also entered on a REDCap database and underwent content analysis using NVivo 12 (QSR International, Melbourne, Australia). Content analysis is a widely used method of qualitative analysis that includes organizing information based on emergent themes from the text and sorting themes into categories to further understand how issues are related.^18^

### Ethical Considerations

Approval for the CLIP Trial was obtained from the Institutional Bioethics Review Boards of Centro de Investigação em Saúde da Manhiça (CISM, CIBS-CISM/038/14), the Mozambique National Bioethics for Health Committee (219/CNBS/14) and the University of British Columbia (UBC, H12-03497). Written informed consent was obtained from households participating in the CLIP Trial.

## RESULTS

### Needs Assessment

During the needs assessment stage, 97 community dialogue sessions were held in 57 neighborhoods involving 2,456 participants between October 2015 and March 2016. These sessions involved discussion on the acceptability and feasibility of the community transport program, considering specific local conditions. Over half (35/57) of the communities engaged in the dialogues showed interest in the program and were invited to the subsequent step of establishing a community-based transport program.

### Community Mobilization and Preparation for Implementation

**FIGURE 2:**
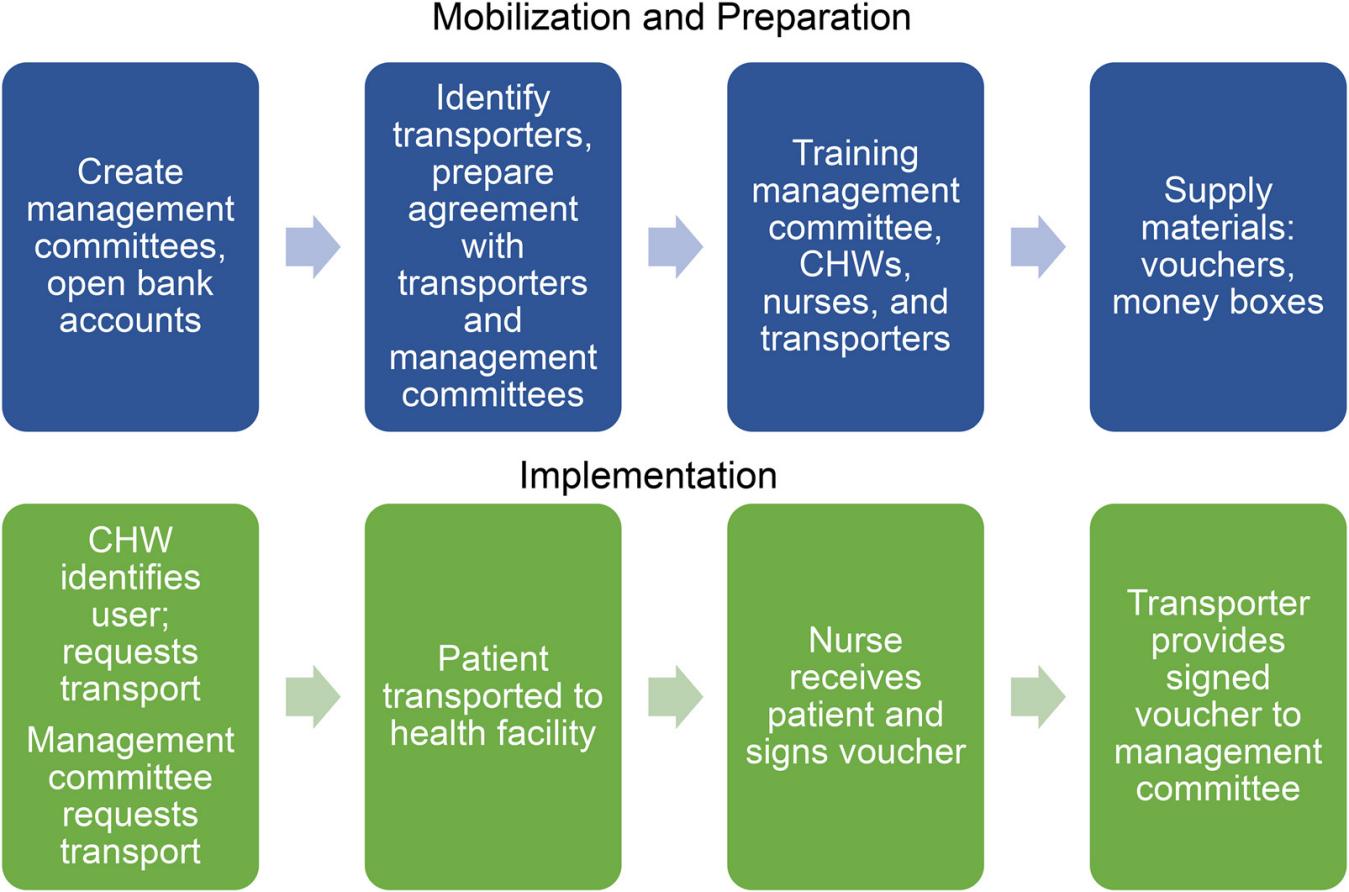
Process of Preparing and Implementing the Community Transport Program to Improve Access to Emergency Obstetric Care, Southern Mozambique

Figure 2 shows the steps involved with mobilizing the community for implementing the community transport program.

**Create management committees:** Initially, community health committees and primary health facilities were responsible for fund management. However, after consultation with the provincial health directorate and community members, it was requested that the fund be managed by people chosen by the community. Consequently, each neighborhood chose 3 to 4 individuals to be in a management committee to manage funds and implement the transport program. Communities were supported by CLIP trial staff in the creation of the committee. Committees were responsible for opening a bank account, collecting money from contributing households, depositing and withdrawing funds, and monitoring use of the fund. All committee members had to be literate, have adequate identification documentation, and be trusted by the community. Literacy and identification documentation were essential requirements for opening up a bank account.

Funds were managed by management committees comprised of 3-4 people chosen by the community.

**Open bank accounts:** When opening bank accounts, the study team provided support, including transport, to the management committees. Establishing bank accounts for a specific community activity required complex and lengthy procedures, which entailed multiple trips to the banks located in the urban centers. Issues encountered included the cost of preparing documentation like personal identification, personal income taxes declaration, and other bank account requirements. If a member could not read or write, they had to obtain a power of attorney for someone else to sign on their behalf.

**Prepare agreements with local transporters and management committees:** Each participating neighborhood created a list of all local transporters including all community members who owned a means of transport (vehicles and tractors). Some communities required particular modes of transport, such as Calanga where a tractor was necessary to travel over sand. A meeting was held with each potential transporter to prepare a transportation agreement and to negotiate remuneration for the services provided. Transporters were sensitized about the need and benefits of the program and encouraged to provide discounted travel costs as a contribution and support to their communities. As a result of the sensitization, it was agreed that remuneration would cover only the cost of fuel. Costs were reduced on average US$5.12 per journey (Supplement 2 Table S2).

The CLIP Trial provided start-up funds. As an institutional procedure, an agreement was established between the funder (Manhiça Health Research Center) and the management committees representing each participating community.

In the 35 neighborhoods, CLIP staff conducted community involvement meetings to explain the steps to prepare for the program as follows.

**Provide training:** Training was provided to committee management members, CHWs, nurses, and transporters on the management and procedures of the transport program including assessing eligibility for beneficiaries based on emergency conditions and filling out transport vouchers. Building on the existing referral slip system used by CHWs, transport vouchers served to confirm the use of transport and facilitate payment. The voucher recorded the date, CHW signatures, nurse's name who assisted the patient, and transporter's name. During training sessions, roles and responsibilities were clarified.

- Management committee: The local management committees comprised entrusted community members to manage the transport fund. In addition, the committee provided oversight to ensure the fund was being used appropriately for eligible health emergencies (Box).
- Local transport providers: Private transport providers in the communities were engaged to transport patients in 1-way trips to the nearest health facility when requested by the management committees.
- CHWs identified emergencies and eligible patients for transport (Box).
- Nurses working at local health facilities received transported patients and confirmed transport vouchers that patients carried.

**Distribute supply materials:** Start-up materials were distributed to management committees. These supplies included boxes to store small amounts of money for emergencies, vouchers for patients, and monitoring forms to log all transportation events.

BOX **Eligibility for Transport Program Use**

- A resident of the neighborhood
- A member of a household that contributed to the community transport fund
- An obstetric emergency, confirmed by community health worker (CHW)
- Serious medical condition, confirmed by CHW (pregnant and postpartum woman, children, and the elderly were prioritized)

### Program Implementation and Launch

After the preparation process, a round of meetings were held to disseminate the key contacts, including the local transporters. These meetings also launched the program and the mutually agreed upon procedures (Figure 2).

Meeting participants were mostly women (81%), between age 26 and 50 years (Table). Fifty-four percent of the attendees were married or cohabiting with their partners, and 39% reported to be Christian. Meeting participants had a low level of education, 22% never studied, and 22% had completed only the primary level of education.

**TABLE.** Community Mobilization Meeting Participant Characteristics, Southern Mozambique

	Participants in Interested Neighborhoods (N=1002)	Participants in Implementing Neighborhoods (N=434)
**Gender, no. (%)**		
Female	811 (80.9)	352 (81.1)
Male	191 (19.1)	82 (18.9)
**Age**		
Age, median (interquartile range)	35.0 (25.0, 49.0)	35.00 (27.0, 48.0)
≤ 20 years, no. (%)	140 (14.0)	56 (12.9)
21–25 years, no. (%)	137 (13.7)	45 (10.4)
26–35 years, no. (%)	246 (24.6)	125 (28.8)
36–50 years, no. (%)	248 (24.8)	125 (28.8)
≥ 51 years, no. (%)	231 (23.1)	83 (19.1)
**Education level, no. (%)**	
No education	222 (22.2)	116 (26.7)
Primary level	224 (22.4)	104 (24.0)
Secondary level	46 (4.6)	9 (2.1)
Higher education	5 (0.5)	0 (0.0)
Don't know	1 (0.1)	0 (0.0)
Other	504 (50.3)	205 (47.2)
**Marital status, no. (%)**	
Married/marital union	545 (54.4)	217 (50.0)
Divorced/separated	19 (1.9)	0 (0.0)
Single	358 (35.7)	171 (39.4)
Widowed	71 (7.1)	46 (10.6)
Other	9 (0.9)	0 (0.0)
**Religion, no. (%)**		
Christian–Protestant/Evangelic	452 (45.1)	246 (56.7)
Catholic	239 (23.9)	108 (24.9)
Atheist/Agnostic	38 (3.8)	16 (3.7)
Hindu	1 (0.1)	0 (0.0)
Islamic	1 (0.1)	0 (0.0)
Other	271 (27.0)	64 (14.7)

### Uptake of the Community Transport Program

Of the 35 interested neighborhoods, 9 neighborhoods initially joined the community transport program. Testimonials from beneficiaries were shared at CLIP community engagement meetings. After witnessing the success of the program in those neighborhoods, 5 more neighborhoods requested to join the program. Of these, 4 were added to the program; 1 was unable to join because it did not meet the minimum required community contribution.

Ultimately, 13 neighborhoods implemented the community transport program based on their willingness to participate and ability to contribute financially. This financial contribution was a requirement to consider the program operational in each neighborhood. The contribution amounted to an average of US$0.33 (ranging from US$0.07 to US$0.72) per family per month. Additionally, to support the start-up costs, the CLIP Trial provided seed funds, which ranged from US$35.29 to US$286.79, based on the predicted number of obstetric emergencies within the neighborhood for the duration of the CLIP Trial (Supplement 2, Table S3). These funds were used only for transport; community management committees volunteered their time, and CLIP supported administration of the program.

Each of the 13 neighborhoods received a safety box to keep small amounts of money at hand for emergencies. Additionally, 8 participating neighborhoods successfully opened bank accounts to safely keep larger amounts. There were no neighborhoods that dropped from the program during the study period.

**FIGURE 3:**
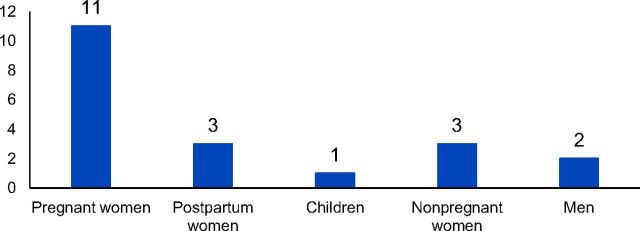
Number of Individuals Transported in the Community Transport Program by Target Group, Southern Mozambique (N=20)

During the implementation of the transport program (from April 2016 to February 2017), the transport funds were utilized on 20 occasions in 10 neighborhoods. A majority of the cases were for obstetric emergencies and 70% of the beneficiaries were pregnant or postpartum women (Figure 3). Although the program was initially designed to support transport for obstetric emergencies, community feedback during implementation led to broadening the scope to include other contributing family members. However, the focus remained on pregnant and postpartum women. No transport events were prevented due to weather conditions.

**FIGURE 4:**
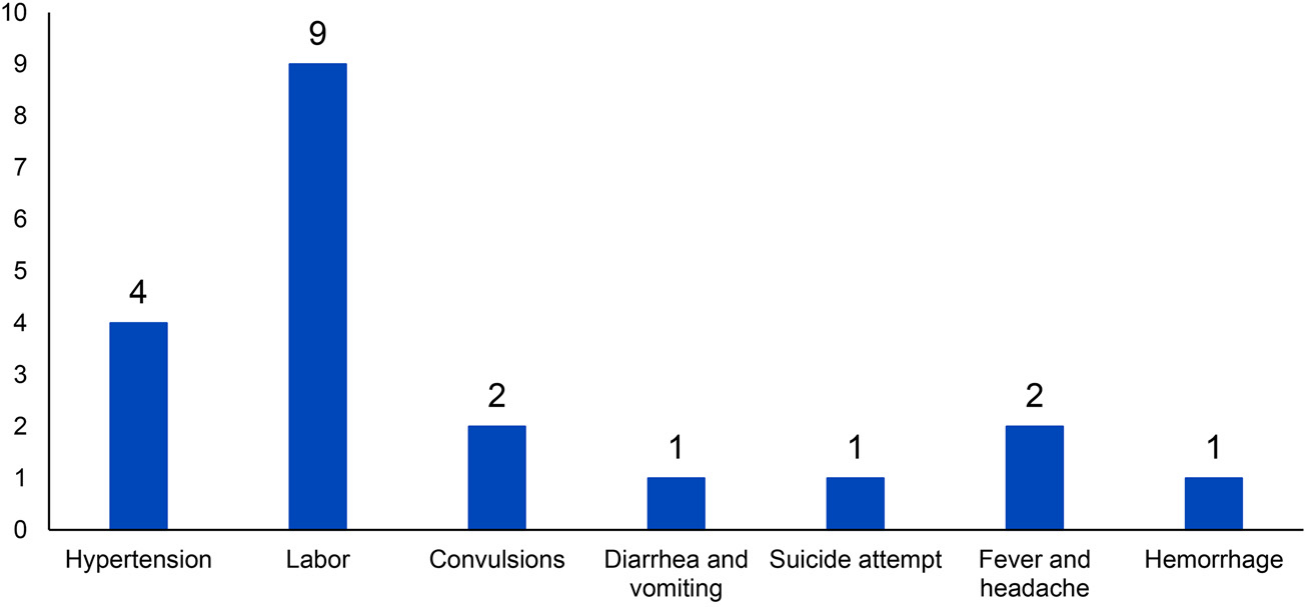
Number of Individuals Who Requested Community Transport by Condition That Triggered Request, Southern Mozambique (N=20)

Transport was provided to 11 pregnant and 3 postpartum women for cases of labor pains, hypertension, convulsions, hemorrhage, a suicide attempt, and fever (Figure 4). There were no maternal deaths registered among those who benefited from the community transport program. Additionally, there was 1 child, 3 nonpregnant women, and 2 men who received transport for health conditions including severe diarrhea, vomiting, and fever.

In all cases, the transporter provided the voucher signed by the nurse to the management committee after the transfer and received the appropriate remuneration.

### Qualitative Data

#### Positive Experiences and Benefits

One pregnant woman began to have contractions in the middle of the night. Early the following morning, her mother-in-law contacted the management committee to request transport. The mother-in-law shared that the transporter arrived quickly and took the woman to the health facility where she was assisted immediately. The woman had a normal delivery, and both mother and baby were well. She was discharged the following day and returned home via minibus paid for by her husband:

*I felt very good about being transported to the hospital by the transport program when I needed, and I would like to encourage the community to continue contributing the money for the funds because it helps people a lot. I hope this project continues to help more people. I'm very grateful to the management committee who called the transporter and if it were not for this I would have given birth at home. I will continue to contribute to the fund because I saw the importance of it.* —Pregnant woman who used community transport system, Chissano, Gaza Province

Another woman suffered from postpartum hypertension. A few days after delivery, she was visited by a CHW who found that she was very weak and not feeling well. The CHW recognized that she was hypertensive and recommended administration of an antihypertensive (methyldopa), seizure prophylaxis (magnesium sulfate), and referral to the nearest health facility as part of the CLIP Trial procedures. The CHW called the local transporter, who arrived quickly and took the woman to the nearest health facility. Upon arrival, the nurse assessed the woman and confirmed the hypertension diagnosis. The woman was later discharged. Because the transporter had already left, the woman paid for a minibus to return home. Notes taken by the facilitator during a general community meeting described how this woman's mother-in-law shared the story with others to encourage participation in the program.

*One mother-in-law said that the transport fund is working because her daughter-in-law was sick after delivery, and the driver had to be called to take her to the nearest health facility. After being taken care of, she improved and is well. All thanks to the transport fund of the community. She stresses that they must continue to contribute for the benefit of the community.* —From a general community meeting, Messano, Gaza Province

Women who benefited from the community transport program often shared positive stories about their experience. Women and families were excited to share their experiences and encourage others to participate in the program. Women and families appreciated the initiative and encouraged others to join the program.

*One of the elders said that this mobilization will be very important for many women … One of the elders spoke of money for transportation in the event of an emergency … They suggested everyone should contribute.* —From a meeting with elders and women of reproductive age in Malehice, Gaza Province

*There is transport that helps people in emergencies, but the price (of transport) is very high, and people who cannot afford the transport use the hand cart to take their relative to the health facility. There are times when a person dies for lack of money to pay for transport.* —From a mobilization meeting with neighborhood chiefs, small business owners, partners, and husbands in Malehice, Gaza Province

#### Challenges to Implementation and Delivery of the Transport Strategy

Community members shared some of the challenges encountered during mobilization and follow-up meetings. Although most neighborhoods were able to develop a list of local transporters, some experienced difficulty collecting this information and had a lack of local transporters.

*The leaders were very happy but said there is only 1 tractor in the community…and the owner uses it on his farm, and it is not possible to take the community to the hospital*. —From a meeting with neighborhood chiefs and small business owners in Calanga, Maputo Province

Additional challenges included the highly bureaucratic process of opening bank accounts, which was limited to those who were literate, had the required identification and documents, and had personal income tax documentation. Because of this process, many community members could not participate in the management committee. From the perspective of CHW supervisors and directors of health facilities, it was important to distance CHWs and nurses from fund management so that there was no interference with the provision of clinical care. Further, there was tension over who benefited from the funds and how it was managed in the community. Men, in particular, highlighted how a community transport fund to support obstetric emergencies would not benefit them.

*The participants complained a lot about the issue of contributing to the fund, considering that the money would only benefit the pregnant women … Some ladies who were present said that they would not contribute because they do not or will not have children, so they would be contributing in vain.* —From a meeting with neighborhood chiefs and small business owners in Malehice, Gaza Province

Challenges to implementation included lack of available transporters, barriers to opening bank accounts that limited participation, and tension on how to manage funds.

Tensions emerged between those who did and those who did not contribute within the same neighborhood.

*The participants were not pleased by the fact that most people are not contributing to the fund.* —From a meeting with community members in Messano, Gaza Province

Furthermore, inconsistent contributions challenged the sustainability of the transport program. Community management committees reported that there were difficulties in collecting contributions. This was sometimes related to the high burden of poverty among the neighborhoods. During implementation, the communities reported suffering from consequences of the droughts and shared that they already had trouble feeding their families and did not have extra funds to contribute.

*As for the emergency transport fund, they said they would like but what hinders is the hunger that has been going on because it has not been easy even to eat.* —From a meeting with husbands and partners in Chaimite, Gaza Province

The ultimate challenge was ensuring a smooth phase-out while motivating communities to continue with the program beyond the timespan of the CLIP trial. During the phase-out visits to each community, community leaders, particularly the heads of administrative posts in Gaza province, showed willingness to supervise the program implementation themselves. One of them, who possessed a vehicle herself, realized only during the phase-out stage that she could have played the role of a transporter, to which the CLIP team's response was to encourage the practice.

## DISCUSSION

This assessment illustrates participative community-based efforts to address the lack of transport from the community to health facilities in rural Mozambique in the view of assuring a continuum of care, which is a gap already identified by formative research and existing literature.^19^ A detailed preparation process with participating communities, local ownership, and leadership was emphasized, along with local resource mobilization to develop community solutions to a local problem. The community transport program supported 14 pregnant and postpartum women from 13 rural and remote neighborhoods to access emergency health services along with 6 other members of the same communities over 10 months of its implementation. These individuals would not otherwise have had access to prompt, adequate health services. The program also encouraged saving funds for health emergencies; strengthened the existing relationship between CHWs and PHC nurses and their respective communities and health facilities; strengthened the capacity for community mobilization; and helped raise awareness about maternal, newborn, and child care with a focus on prevention and prompt health care seeking behaviors and the need for birth preparedness. Health promotion, particularly regarding maternal, newborn, and child health, is already part of the mandate of CHWs and PHC nurses.^20^^–^^22^ Although there was significant community interest and beneficiaries spoke highly of the program, administrative and socioeconomic limitations prohibited rapid expansion.

The program provided access to prompt health services and strengthened the relationship between CHWs and PHC nurses and their communities and health facilities.

Other community transport programs have been tried elsewhere. In Nigeria, Shehu et al. reported on mobilizing a local union of transport providers to provide timely and affordable transport for women with obstetric complications as well as other members of the community for emergency care.^23^ Costs were shared between the research team, who provided seed funding to purchase fuel, and community members, who provided maintenance funding. The Nigerian program transported 29 women with obstetric emergencies over 1 year, and community response was positive as it dramatically reduced waiting times to organize transport (the second delay). The authors concluded that the strategy could support physical and financial access to obstetric care.^23^ Ensor et al. used a quasi-experimental approach to investigate the effect of a complex community-based intervention that included community transport in Zambia.^24^ Working through existing government-established Safe Motherhood Action Groups, community volunteers were trained and provided with locally appropriate transport to health facilities.^24^ The percentage of women who used emergency transport significantly increased by 12.4%–18.7% (*P*<0.001) from under 10 women at baseline to 66–149 with the 10–15 month intervention, depending on different districts.^24^ Although both of these studies were community-based and involved community members, they were not owned by community members. Additionally, the vehicles from the Zambian study were not provided by the community but purchased purposively for the transport program, which may have decreased the sense of local ownership and impaired sustainability.

Organized on the principle of associativism (collective cooperation), which highlights cooperative and collaborative partnership making within communities for collective action to improve conditions,^25^ this program uniquely emphasized community ownership and leadership at all stages. Core values of this initiative included ensuring community consensus and getting members of the community management committee to open bank accounts to keep funds, as well as ensuring solidarity, participation, unity, cooperation, and the focus on common objectives.^25^ The CLIP research team provided technical assistance to the program, but decisions were built by consensus within each community, which allowed for flexibility to local conditions and supported local skill and capacity building for financial management. With the above-mentioned focus on community ownership, management committee members were trained to be able to continue the program after the project.

Core values of this initiative included ensuring community consensus, solidarity, participation, unity, cooperation, and the focus on common objectives.

### Lessons Learned

Lessons learned in designing and implementing the transport program in the CLIP trial in Mozambique included that in many of the cases, successful implementation depended on availability of transport, interest, and willingness of the community to manage the program, as well as affordability of the family contributions. Communities already close to primary health centers, such as Três de Fevereiro, were less inclined to participate. Additionally, communities that did not have close access to a bank had fears on holding large sums of money in neighborhood settings. However, with introduction of mobile money innovations like M-Pesa, these concerns may be lessened. There were fears that CHWs and nurses may inappropriately manage the funds; therefore, community-based management committees were essential. The involvement of community leaders and stakeholders to build trust in the program was critical. Lastly, it was found that very poor communities were least interested in the program, which emphasizes that any individual program needs to be embedded into other poverty reduction strategies. Consequently, advocacy involving the Ministry of Gender, Children and Social Affairs and the Ministry of Transport and Communications in addition to the Ministry of Health, as well as engagement with other partners like nongovernmental organizations is needed. Based on such a cross-sector approach, the government could play a valuable role in providing overarching directives to support coordination of the various poverty reduction and health strengthening programs.

The Community Health Worker Performance Measurement Framework highlights that the ability to facilitate referrals from the community to the health facility while maintaining continuity of care and counter-referrals from the health facility back to the CHWs is essential for effective community health.^9^ While previous research has reported on challenges to referral systems between levels of care in sub-Saharan Africa,^16^ transportation between health facilities did not seem to be a major issue in this study, provided that in Mozambique ambulances only serve to transport people between health facilities.^26^ Community members in this study especially highlighted the lack of transport between the community and primary health facilities, despite the fact that this referral pathway is expected within the framework of the national health services. The national guidelines point to clear referral pathways of patients to and between health facilities and places the CHWs as the interface between the community and the PHC facility in the referral process. However, there is a lack of detail on possible ways to make this process effective and timely, particularly in cases where the mobility of the patient is compromised by distance, physical incapacity to walk to the health facility, and other barriers. This policy gap can serve as an opportunity to fill it in with concrete suggestions based on evidence such as that brought up by the current study. In this way, addition of a community transport element to the existing referral system would not be a burden but a complement to the referral system. Likewise this would not be a drastic addition to the current PHC strengthening program (2017–2023), which already seeks to improve indicators such as institutional deliveries; health facilities providing basic and comprehensive emergency obstetric and newborn care; and trained and active CHWs, all of which could be enhanced by improved referral circuits.^27^^,^^28^

Although national guidelines point to CHWs linking the community to the PHC facility through clear referral pathways, details are lacking on how to make this process cost-effective and timely, particularly for patients in remote areas.

Though some transport challenges persisted, the program implementation had positive impacts including streamlining the process of identifying transport options and collaborators in the community, pre-negotiating travel prices to increase affordability, and raising community attention to the role of transport in reducing delays in health care seeking. Sharing stories of successful facility births and well-being of mothers and babies as a result of the new transport program contributed to building momentum and increased attention to maternal health in the community.

The community transport program developed with the Mozambique CLIP Trial illustrates the importance of transport in ensuring maternal and child health by leveraging an existing referral system. Without access to transport from the community level to health facilities CHW performance and associated health outcomes may be compromised.^19^ During the dissemination of these findings, the Mozambican Ministry of Health officials revealed their interest in adopting a bicycle-ambulance program in other regions of the country. They valued and showed openness to adopt the community ownership and management aspect of the program to support sustainability. At the local level, administrative post chiefs' enthusiasm further served as encouragement to support advocacy for programs of this nature to be replicated, yet adjusted to local realities also aligned with the much commended decentralization of some aspects of governance in Mozambique.^28^

A strength of the study was the close working relationship between the CLIP Trial staff and the community and respective community leaders, cultivation of leadership and collaboration among the local management committees, collaboration of multiple local stakeholder groups, and the constant feedback which allowed for adjustments to the program. Future areas for exploration include understanding the impacts of seasonality on referral patterns, the added value of transport programs for back-referrals, and the specific needs of extremely underserved communities.

### Limitations

A limitation of the study was the relatively low participation of eligible neighborhoods (13 of 57), in part due to the complex and lengthy procedures to open a bank account and the short study period. This may mean that the participating communities were not representative of the region and limits the transferability of our findings through some of the broader challenges highlighted in this study regarding transport infrastructure that are common across the region.^15^ Although the CLIP trial activities in the community relied only on CHWs, communities are also served by matrons, traditional birth attendants, and traditional healers.^29^ To increase the number of beneficiaries, the transport program would need to reach these health care providers to cover the wider community.

Data were collected by staff who supported the facilitation of the program, thus there may be some bias to reporting positive results, as often is the criticism of action research study design. Because the transport program was embedded in the CLIP Trial, there was overlap in the staff for training and supervision, thus there is limited information on the specific costing of external management to start the program. Furthermore, the time between transport request and when it was made available was not systematically recorded, though all were able to reach care within the recommended 4 hours for urgent referrals recommended in the CLIP Trial.

Another limitation of the study was that the participation in the program depended on local capacity to raise money and fulfill the requirements for the administrative processes. With rates of poverty between 12% (Maputo Province) and 44% (Gaza Province),^30^ this could explain the observed low contributions even when community members were highly interested in the transport program. Families needed to prioritize the multitude of problems faced in their daily lives. The immediate challenge of securing adequate food for their households shared by some participants took priority over contributions to a community fund for preventative measures. This hindered both the ability for neighborhoods to join the program and the sustainability of the fund among participating neighborhoods. As a result, whole communities, although highly motivated, were denied joining this program due to lack of capacity to provide the minimum contribution.

Additionally, some communities had poor road access and lack of transport options, which are key pillars for the program to take effect. Previous mapping of access to maternal health services found that approximately 85% of the roads are unpaved, hard to traverse in the rainy season and unpassable during seasonal flooding.^15^ The extreme case was Calanga administrative post, which is very sandy with poor quality roads and heavily affected by the rains. As illustrated in the results, community leaders from Calanga expressed interest in the program but were also quick to report the lack of transport options available and poor road infrastructure that hindered the feasibility of the program.

While the community transport program was developed to help mitigate challenges in accessing care, emergency transport provided was often 1-way to health facilities, which could have also contributed to some of the nonadherence to the program. Faced with limited funds, community management committees decided to prioritize emergency transport to PHC facilities and not the other way, reasoning that once the person had been assisted at the facility, their health either would have stabilized, therefore the return home would not be as dramatic, or they would require further referral to higher-level facilities by ambulance instead. However, paying for transport back to the community emerged as a potential barrier that should be further investigated and addressed.

Further, communities that faced challenges implementing the transport program were often those with a low density of health facilities.^15^ This also raises the importance of assessing health services coverage and readiness to meet the potential higher demand for health services created by improvements in the referral systems from communities to health facilities. In this study, health facilities managed to provide prompt assistance in response to this demand, but this may not be always the case in similar settings.

## CONCLUSION

The gap created by lack of transport within the existing referral system between the community and the PHC facility poses a barrier to access to emergency obstetric care in southern Mozambique. In strengthening capacities for community health and the role of CHWs, it is crucial to encourage local transport programs and transport infrastructure in poorly-resourced communities to support access and engagement with health systems. The community transport program developed as part of the Mozambique CLIP Trial illustrated that it was feasible to implement such a program to address not only emergency obstetric care needs but also health problems. The program was implemented with no external input of vehicles, fuel, personnel, or maintenance and with minimum requirements within the communities. The transport program facilitated several emergency cases that would not have otherwise had prompt access to health care.

However, there were numerous challenges such as appropriate members of the community to manage the fund, unfavorable terrain, few available transport options, and poverty. Addressing these challenges is recommended to ensure the strategy is sustainable. This includes increasing community awareness and leadership to maximize demand and decrease the cost per family, establishing agreements with banking systems that make processes more flexible, and raising awareness and interest among potential implementing partners in health, public works, and nongovernmental organizations to support community efforts and infrastructure. Among such partners, there is a need to assign or create capacity to perform rapid needs assessments before implementation to account for communities' specific needs with particular attention to existing infrastructure, socioeconomic inequities within communities, gender and cultural norms, existing knowledge and awareness about care seeking, as well as geographical and climatic challenges.

## Supplementary Material

20-00511-Mungumabe-Supplement2.pdf

20-00511-Mungumabe-Supplement1.pdf

## Translation

**Em português**

**Implementação de uma estratégia de transporte comunitário para reduzir atrasos na procura de cuidados obstétricos na zona rural de Moçambique**

**Mensagem chave:** Incentivar programas de transporte local e infraestrutura de transporte em comunidades com poucos recursos pode ajudar a melhorar o acesso da comunidade e fortalecer o envolvimento com os sistemas de saúde. A mobilização de recursos e liderança da comunidade para implementar um esquema de transporte baseado na comunidade na zona rural de Moçambique para apoiar encaminhamentos para unidades de saúde pode ajudar a melhorar os resultados de saúde materno-infantil.

**Principais achados**

- Foi viável implementar um programa de transporte de base comunitária em zonas rurais sem qualquer recurso externo em termos de veículos, combustível, pessoal ou manutenção. Contudo, a falta de transportadores disponíveis, barreiras para cumprir os requisitos para a abertura e gestão de contas bancárias, as iniquidades na acessibilidade dos custos do programa e tensões relativas a opiniões sobre a forma de gerir os fundos foram os desafios identificados na implementação e que requerem apoio adicional.
- Os impactos positivos da implementação do programa incluíram a racionalizaçäo do processo de identificação de opções de transporte e de colaboradores na comunidade, a pré-negociação dos custos das viagens para aumentar a acessibilidade dos mesmos e uma comunidade prestando mais atenção ao papel do transporte na redução dos atrasos na procura de cuidados de saúde e sensibilizada para o reconhecimento precoce dos sinais de perigo e a rápida procura de cuidados

**Principais Implicações**

- O nosso programa de transporte baseado na comunidade enfatizou de forma única a apropriação e liderança da comunidade em todas as suas fases. As decisões foram construídas por consenso no seio de cada comunidade, o que permitiu flexibilidade perante as condições locais e apoiou o desenvolvimento de competências e capacidades locais para a gestão financeira.
- A implementação dos esquemas de transporte não pode estar alheia aos esforços de fortalecimento do sistema de saúde em geral. Este esquema utilizou mecanismos de referência existentes, em vez de criar processos inteiramente novos, de modo a assegurar a sustentabilidade.

**Resumo**

**Introdução:** Atrasos resultantes de longas distâncias percorridas para as unidades sanitárias, infra-estruturas rodoviárias deficitárias e falta de opções de transporte acessíveis contribuem para o peso das mortes maternas em Moçambique. Este estudo teve como objectivo avaliar a implementação e adopção de um esquema de transporte inovador baseado na comunidade para melhorar o acesso aos cuidados obstétricos de emergência no sul de Moçambique.

**Métodos:** De Abril de 2016 a Fevereiro de 2017 foi implementado um esquema de transporte como parte do ensaio de Intervenções a Nível Comunitário para a Pré-eclâmpsia (CLIP), que visava avaliar a redução da mortalidade e morbilidade materna e perinatal em 20% nos conglomerados de intervenção nas províncias de Maputo e Gaza, Moçambique através do envolvimento dos Agentes Polivalentes Elementares na identificação e encaminhamento das mulheres grávidas e puérperas em risco. O esquema de transporte foi desenvolvido com base numa avaliação participativa das necessidades da comunidade. Durante a implementação do esquema de transporte foram recolhidos dados demográficos, das condições que requeriam transporte, os meios de transporte utilizados, os itinerários e os desfechos de cada caso. Os dados foram introduzidos numa base de dados em REDCap.

**Resultados:** Cinquenta e sete bairros participaram na avaliação das necessidades; dos quais, 13 (23%) implementaram o esquema de transporte. Os bairros foram seleccionados com base na demonstração de interesse e capacidade de contribuir financeiramente para o esquema (US$0,33 por família, por mês). Em cada bairro seleccionado foi criado um comité de gestão comunitário que recebeu formação em gestão financeira de pequena escala e acesso aos instrumentos para monitoria do funcionamento do esquema. Vinte pessoas de 9 bairros beneficiaram-se do esquema de transporte, das quais 70% eram mulheres grávidas ou puérperas.

**Conclusão:** Estes resultados demonstram que foi viável implementar um esquema de transporte baseado na comunidade, sem qualquer contributo externo em veículos, combustível, pessoal e manutenção. Contudo, o custo e a falta de opções de transporte aceitáveis continuam a dificultar o acesso a serviços de saúde obstétricos e a capacidade de seguimento atempado. Ao reforçar as capacidades dos Agentes Polivalentes Elementares para prestar assistência e encaminhar rapidamente os casos de emergência, é crucial encorajar esquemas e infra-estruturas locais de transporte em comunidades com recursos mínimos para apoiar o acesso e fortalecer o envolvimento com os sistemas de saúde.

**Peer Reviewed**
