# Supplementary material for: Implementation of a Community Transport Strategy to Reduce Delays in Seeking Obstetric Care in Rural Mozambique
Source: Glob Health Sci Pract. 2021 Mar 15;9(Suppl 1):S122–36. doi: 10.9745/GHSP-D-20-00511 (PMC7971369; doi:10.9745/GHSP-D-20-00511)
Supplement: 20-00511-Mungumabe-Supplement2.pdf [file 20-00511-Mungumabe-Supplement2.pdf]

Supplement to: Amosse F, Boene H, Kinshella M, et al. Implementation of a community transport strategy to reduce delays in seeking obstetric care in rural Mozambique. *Glob Health Sci Pract*. 2021;9(Suppl 1). https://doi.org/10.9745/GHSP-D-20-00511

**Table S2. Transport costs before and during the program in Mozambique Metical (USD*)**

| Cluster | Neighborhoods | Cost of transport before | Cost of transport after negotiation | Savings with the transport program |
|---|---|---|---|---|
| Messano | Neighborhood A | 250 ($3.48) | 200 ($2.78) | 50 ($0.70) |
| | Neighborhood B | 500 ($6.96) | 500 ($6.96) | 0 |
| | Neighborhood C | N/A | 400 ($5.57) | N/A |
| | Neighborhood D | N/A | 400 ($5.57) | N/A |
| Chissano | Neighborhood E | 1500 ($20.88) | 500 ($6.96) | 1000 ($13.92) |
| | Neighborhood F | 1000 ($13.92) | 500 ($6.96) | 500 ($6.96) |
| | Neighborhood G | 500 ($6.96) | 300 ($4.18) | 200 ($2.78) |
| Chaimite | Neighborhood H | 1000 ($13.92) | 675 ($9.40) | 325 ($4.52) |
| Malehice | Neighborhood I | 750 ($10.44) | 500 ($6.96) | 250 ($3.48) |
| | Neighborhood J | N/A | 1000 ($13.92) | N/A |
| Calanga | Neighborhood K | 850 ($11.83) | 500 ($6.96) | 350 ($4.87) |
| | Neighborhood L | 750 ($10.44) | 200 ($2.78) | 500 ($6.96) |
| | Neighborhood M | 750 ($10.44) | 200 ($2.78) | 500 ($6.96) |

** 1 MZN = 0.01392 USD*

**Table S3. Cumulative contributions by neighborhood in Mozambique Metical (USD*)**

| Cluster | Neighborhood (bairros) | Initial start-up fund | Cumulative of community contributions | Money used | Number of beneficiaries |
|---|---|---|---|---|---|
| Messano | Neighborhood A | 17,119.86 ($238.31) | 500 ($6.96) | 600 ($8.35) | 3 |
| | Neighborhood B | 10,000 ($139.20) | 450 ($6.26) | 1000 ($13.92) | 2 |
| | Neighborhood C | 10,101.48 ($140.61) | 650 ($9.05) | 400 ($5.57) | 1 |
| | Neighborhood D | 2,440.63 ($33.97) | 500 ($6.96) | N/A | |
| Chissano | Neighborhood E | 12,672.00 ($176.39) | 4500 ($62.64) | 2,000.00 ($27.84) | 4 |
| | Neighborhood F | 13,875.84 ($193.15) | 200 ($2.78) | 500 ($6.96) | 1 |

Supplement to: Amosse F, Boene H, Kinshella M, et al. Implementation of a community transport strategy to reduce delays in seeking obstetric care in rural Mozambique. *Glob Health Sci Pract*. 2021;9(Suppl 1).
https://doi.org/10.9745/GHSP-D-20-00511

| | | | | | |
|---|---|---|---|---|---|
| | Neighborhood G | 19,831.68 ($276.06) | 350 ($4.87) | 300 ($4.18) | 1 |
| Chaimite | Neighborhood H | 10,137.60 ($141.12) | 480 ($6.68) | 2700 ($37.58) | 4 |
| Malehice | Neighborhood I | 10,406.46 ($144.86) | 1800 ($25.06) | 500 ($6.96) | 1 |
| | Neighborhood J | 6,915.11 ($96.26) | 1700 ($23.66) | 1000 ($13.92) | 1 |
| Calanga | Neighborhood K | 5,385.00 ($74.96) | 560 ($7.80) | 500 ($6.96) | 1 |
| | Neighborhood L | 5,068.80 ($70.56) | 650 ($9.05) | N/A | |
| | Neighborhood M | 10,137.06 ($141.11) | 500 ($6.96) | 200 ($2.78) | 1 |
| Total | | **134,091.52 ($1,866.55)** | **12,840.00 ($178.73)** | **8,800.00 ($122.50)** | **20** |

* 1 MZN = 0.01392 USD
