# Supplementary material for: Implementation of a Community Transport Strategy to Reduce Delays in Seeking Obstetric Care in Rural Mozambique
Source: Glob Health Sci Pract. 2021 Mar 15;9(Suppl 1):S122–36. doi: 10.9745/GHSP-D-20-00511 (PMC7971369; doi:10.9745/GHSP-D-20-00511)
Supplement: 20-00511-Mungumabe-Supplement1.pdf [file 20-00511-Mungumabe-Supplement1.pdf]

Supplement to: Amosse F, Boene H, Kinshella M, et al. Implementation of a community transport strategy to reduce delays in seeking obstetric care in rural Mozambique. *Glob Health Sci Pract*. 2021;9(Suppl 1).
https://doi.org/10.9745/GHSP-D-20-00511

Table S1. CLIP Mozambique Working Group

**CLIP Mozambique Working Group**	
**First and middle names**	**Last names**
Felizarda	Amose
Ana Ilda	Biz
Rogério	Chiaú
Silvestre	Cutana
Paulo	Filimone
Marta	Macamo
Sónia	Maculuve
Ernesto	Mandlate
Analisa	Matavele
Sibone	Mocumbi
Dulce	Mulungo
Zefanias	Nhamirre
Ariel	Nhancolo
Cláudio	Nkumbula
Vivalde	Nobela
Rosa	Pires
Faustino	Vilanculo
Rahat N	Qureshi
Sana	Sheikh
Zahra	Hoodbhoy
Imran	Ahmed
Amjad	Hussain
Javed	Memon
Farrukh	Raza
Mrutunjaya B	Bellad
Shivaprasad S	Goudar
Ashalata A	Mallapur
Shashidhar G	Bannale
Umesh S	Charantimath
Keval S	Chougala
Richard J	Derman
Vaibhav B	Dhamanekar
Narayan V	Hoonungar
Anjali M	Joshi
Namdev A	Kamble
Chandrasekhar	Karadiguddi
Geetanjali M	Katageri
Avinash J	Kavi
Gudadayya S	Kengapur
Bhalachandra S	Kodkany

Supplement to: Amosse F, Boene H, Kinshella M, et al. Implementation of a community transport strategy to reduce delays in seeking obstetric care in rural Mozambique. *Glob Health Sci Pract*. 2021;9(Suppl 1). https://doi.org/10.9745/GHSP-D-20-00511

**CLIP Mozambique Working Group**	
**First and middle names**	**Last names**
Uday S	Kudachi
Sphoorthi S	Mastiholi
Geetanjali I	Mungarwadi
Umesh Y	Ramadurg
Amit P	Revankar
Olalekan O	Adetoro
John O	Sotunsa
Sharla K	Drebit
Chirag	Kariya
Mansun	Lui
Diane	Sawchuck
Ugochi V	Ukah
Mai-Lei	Woo Kinshella
Shafik	Dharamsi
Guy A	Dumont
Tabassum	Firoz
Ana Pilar	Betrán
Susheela M	Engelbrecht
Veronique	Filippi
William A	Grobman
Marian	Knight
Ana	Langer
Simon A	Lewin
Gwyneth	Lewis
Craig	Mitton
Nadine	Schuurman
James G	Thornton
France	Donnay
Kelly	Pickerill
**CLIP Trial Working Group**	
Esperança Sevene, Eusébio Macete, Khátia Munguambe, Charfudin Sacoor, Anifa Vala, Helena Boene, Felizarda Amose, Rosa Pires, Zefanias Nhamirre, Marta Macamo, Rogério Chiaú, Analisa Matavele, Faustino Vilanculo, Ariel Nhancolo, Silvestre Cutana, Ernesto Mandlate, Salésio Macuacua, Quinhas Fernandes, Rosa Marlene Cuco, Cassimo Bique, Sibone Mocumbi, Emília Gonçálves, Sónia Maculuve, Ana Ilda Biz, Dulce Mulungo, Orvalho Augusto, Paulo Filimone, Vivalde Nobela, Corsino Tchavana, Cláudio Nkumbula<br>Jeffrey Bone, Dustin T Dunsmuir, Sharla K Drebit, Chirag Kariya, Mai-Lei Woo Kinshella, Tang Lee, Jing Li, Mansun Lui, Beth A Payne, Kelly Pickerill, Diane Sawchuck, Sumedha Sharma, Domena K. Tu, Marianne Vidler, Ugochi V Ukah, Laura A Magee, Peter von Dadelszen	
**CLIP Trial Adjudication Committee**	
Nafissa Osman, Cassimo Bique, Natercia Fernandes, Betuel Sigauque Raquel Gonzalez	

Supplement to: Amosse F, Boene H, Kinshella M, et al. Implementation of a community transport strategy to reduce delays in seeking obstetric care in rural Mozambique. *Glob Health Sci Pract*. 2021;9(Suppl 1). https://doi.org/10.9745/GHSP-D-20-00511

**CLIP Mozambique Working Group**	
**First and middle names**	**Last names**
**CLIP Trial Steering Committee**	
J Mark Ansermino, Ana Pilar Betrán, Richard Derman, Shafik Dharamsi, France Donnay, Sharla Drebit, Guy Dumont, Susheela M. Engelbrecht, Veronique Fillipi, Tabassum Firoz, William Grobman, Marian Knight, Ana Langer, Simon Lewin, Gwyneth Lewis, Craig Mitton, Nadine Schuurman, Andrew H Shennan, Joel Singer, Jim Thornton, Hubert Wong	
**CLIP Trial Executive Committee**	
Olalekan Adetoro, Mrutunjaya M Bellad, Zulfiqar Bhutta, Peter von Dadelszen, Shivaprasad S Goudar, Jerker Liljestrand, Laura A Magee, Ashalata Mallapur, Khátia Munguambe, Beth Payne, Rahat Qureshi, Charfudin Sacoor, Esperança Sevene, Sumedha Sharma, John Obafemi Sotunsa, Marianne Vidler	
**CLIP Data Safety and Monitoring Board**	
Romano Nkumbwa Byaruhanga, Brian Darlow, Eileen Hutton, Mario Merialdi, Lehana Thabane	
